# Branched-Chain Amino Acid Intake and Risk of Incident Type 2 Diabetes: Results from the SUN Cohort

**DOI:** 10.3390/biomedicines13102561

**Published:** 2025-10-21

**Authors:** Víctor de la O, Telmo Bretos-Azcona, Francisco Javier Basterra-Gortari, Carmen de la Fuente-Arrillaga, Miguel Ruiz-Canela, Miguel Ángel Martínez-González, Maira Bes-Rastrollo

**Affiliations:** 1 Faculty of Health Sciences, International University of La Rioja (UNIR), 26006 Logroño, Spain; victor.delao@nutricion.imdea.org; 2 Nutritional Control of the Epigenome Group, Precision Nutrition and Obesity Program, IMDEA Food, Campus of International Excellence Universidad Autónoma de Madrid + Spanish National Research Council, 28049 Madrid, Spain; 3 Department of Preventive Medicine and Public Health, School of Medicine, University of Navarra, 31008 Pamplona, Spain; tbretosazco@alumni.unav.es (T.B.-A.); fj.basterra.gortari@navarra.es (F.J.B.-G.); cfuente@unav.es (C.d.l.F.-A.); mcanela@unav.es (M.R.-C.); 4 Department of Endocrinology and Nutrition, Hospital Universitario de Navarra, Universidad Pública de Navarra, 31008 Pamplona, Spain; 5 IdiSNA, Instituto de Investigación Sanitaria de Navarra, 31008 Pamplona, Spain; 6 Biomedical Research Centre Network on Obesity and Nutrition (CIBERobn), Physiopathology of Obesity and Nutrition, Institute of Health Carlos III, 28029 Madrid, Spain; 7 Department of Nutrition, Harvard T.H. Chan School of Public Health, Boston, MA 02115, USA

**Keywords:** branched-chain amino acids, BCAA, type 2 diabetes mellitus, prospective cohort, energy-adjusted intake, Mediterranean diet, dietary assessment, nutritional epidemiology, SUN Project, hazard ratios

## Abstract

**Background/Objectives**: While many studies have explored the association between circulating branched-chain amino acids (BCAAs) and type 2 diabetes mellitus (T2DM), evidence on the prospective relationship between dietary BCAA intake and T2DM risk remains limited. We aimed to explore this relationship—both total and by dietary source—in a Mediterranean cohort. **Methods**: We used data from the SUN Project, a prospective and dynamic cohort of Spanish university graduates initiated in 1999. Dietary intake was assessed with a validated 136-item food frequency questionnaire at baseline and at 10 years. BCAA intake (valine, leucine, isoleucine) was estimated using the USDA amino acid database and adjusted for energy intake by the residual method. Participants were followed biennially through questionnaires to identify incident T2DM cases, confirmed by a supplementary questionnaire and medical report, following the ADA diagnostic criteria. Cox proportional hazards models were used to estimate hazard ratios (HRs) and 95% confidence intervals (CIs), adjusting for potential confounders across four multivariable models. BCAA intake was modeled both categorically (tertiles) and continuously (per 0.5% energy or 5 g/day increase). Analyses were stratified by age and recruitment period. **Results**: After exclusions, 20,154 participants were included (mean follow-up: 14.67 ± 5.8 years), with 220 incident T2DM cases identified. For each 0.5% energy increment intake from BCAA, there was no association with T2DM (adjusted HR: 1.01; 95% CI: 0.69–1.20). Among men, the adjusted HR was 0.91, 95% CI: 0.69–1.20. Among women, it was 1.40, 95% CI: 0.94–2.09. In the overall cohort, higher BCAA intake showed a non-significant inverse association with the T2DM risk when comparing extreme tertiles (HR = 0.81; 95% CI: 0.48–1.37), which strengthened when repeated dietary measures were considered (HR = 0.70; 95% CI: 0.46–1.06, *p*-trend = 0.06). Analyses by BCAA sources (animal vs. plant) and stratified by sex, weight status, and age did not reveal consistent patterns, though exploratory findings suggested potential effect modification by sex and adiposity. Sensitivity analyses confirmed the lack of robust associations, with some subgroup-specific signals being limited by low event numbers and wide CIs. **Conclusions**: Given the power limitations and the modest, non-significant associations observed, these findings should be considered preliminary evidence that may help guide future research on the role of dietary BCAAs in glucose metabolism and diabetes risk.

## 1. Introduction

Type 2 diabetes mellitus (T2DM) is a growing global health concern largely driven by insulin resistance (IR), a multifactorial condition influenced by diet, physical activity, and body composition. Among emerging metabolic factors, branched-chain amino acids (BCAAs)—valine, leucine, and isoleucine—have garnered attention for their potential role in the development and progression of IR and T2DM. These are essential amino acids that comprise approximately 35% of muscle proteins. Around 50% of dietary BCAAs are catabolized in skeletal muscle rather than the liver, unlike other amino acids [1]. Their primary dietary sources include dairy products, meat, fish, eggs, legumes, nuts, and whole grains. Due to their bypass of hepatic metabolism, BCAAs rapidly enter the bloodstream, becoming available to peripheral tissues. Once converted into ketoacids, they can be further oxidized by the liver for energy production [2]. BCAA supplementation may enhance aerobic performance by preserving glycogen stores and stimulating muscle protein synthesis, particularly via leucine-mediated mTOR signaling [2]. In specific populations, such as older adults with T2DM, BCAA supplementation has shown benefits in terms of muscle strength without detrimental effects on IR or renal function, and it may also improve mood [3].

Several studies have reported elevated circulating BCAAs in individuals with obesity and IR [4]. These amino acids have been proposed as early biomarkers for T2DM diagnosis and potential therapeutic targets [5,6]. Increased blood concentrations of BCAAs have been consistently observed in both diabetic and prediabetic individuals [6]. Moreover, higher BCAA levels have been associated with a greater risk of future T2DM onset [5,6,7,8,9,10,11].

The most widely accepted mechanism involves BCAA-induced dysregulation of the mTOR signaling pathway, which contributes to IR [5,9,11]. However, this mechanism is not fully understood. Other studies have found that enhanced BCAA catabolism in obese individuals is also associated with IR, with certain BCAA metabolites outperforming free fatty acids in predicting the T2DM risk [6,11]. The relationship between BCAAs and T2DM may be bidirectional. In early T2DM pathogenesis, elevated BCAA levels may be driven by adiposity, altered hepatic metabolism, and the gut microbiome. Notably, some genetic variants linked to increased T2DM risk also correlate with higher BCAA levels, reinforcing their value as biomarkers [12]. Conversely, elevated BCAAs may also contribute directly to disease progression via various metabolic pathways. While short-term high-protein diets (<6 months) may aid in glycemic control through weight loss, long-term adherence may worsen IR. Diets rich in BCAAs over 4–8 weeks do not appear to impair insulin sensitivity per se, although weight loss remains a key modulator [11]. Meta-analyses of case-control studies support a positive association between high BCAA intake and T2DM risk [13]; however, the findings from prospective cohort studies are less consistent and often limited by confounding factors [8].

Although multiple studies have explored the role of circulating BCAAs in cardiometabolic disease prediction, few have assessed the long-term impact of dietary BCAA intake on incident T2DM in large cohorts. Therefore, we aim to address this gap using data from the prospective SUN (*Seguimiento Universidad de Navarra*) cohort.

## 2. Materials and Methods

### 2.1. Study Population

The SUN Project is a prospective and dynamic cohort study initiated in Spain in 1999. Its main objective is to assess the impact of dietary patterns and lifestyles on health outcomes [14]. Currently, the database includes data up to 30 April 2024. The cohort consists primarily of university graduates, including health professionals and alumni from the University of Navarra. Participants are invited via postal or electronic mail and provide information through validated questionnaires at baseline and every two years thereafter. These questionnaires collect data on sociodemographics, lifestyle, dietary habits, and medical history. A specific questionnaire is also administered at 10 years to update the dietary information. Participation is voluntary, and the baseline questionnaire is considered informed consent, as approved by the institutional ethics committee. The study complies with the Declaration of Helsinki and was approved by the Research Ethics Committee of the University of Navarra (2001/30).

**Figure 1:**
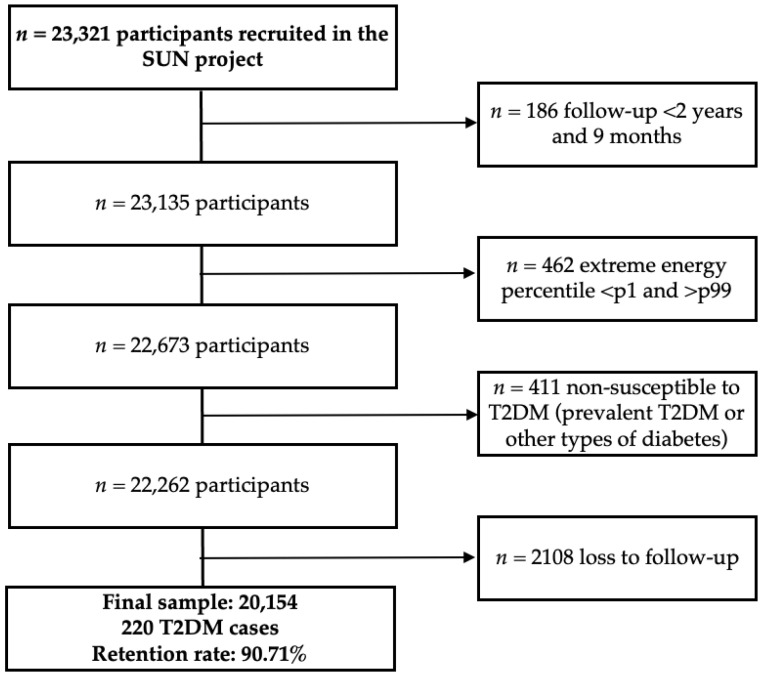
Flowchart of participants in the SUN cohort. Abbreviations: p, percentile, T2DM, type 2 diabetes mellitus.

In 30 April 2024 a total of 23,321 participants had completed the baseline questionnaire. Participants recruited after 30 July 2021 (*n* = 186) were excluded to ensure a minimum follow-up of 2 years. After excluding participants, extreme total energy intake (<1st and >99th percentiles, *n* = 462), prevalent diabetes or non-T2DM cases at baseline (*n* = 411), and loss to follow-up (*n* = 2107), a total of 20,154 participants remained for analysis, among whom 220 incident T2DM cases were identified (Figure 1). The cohort retention rate was 90.71%.

### 2.2. Exposure Assessment: Branched-Chain Amino Acids

Dietary intake was assessed at baseline and at 10 years using the validated semi-quantitative Food Frequency Questionnaire (FFQ), which includes 136 food items (24). The total energy intake (kcal/day), as well as the intake of total protein (g/day), carbohydrates (g/day), total fat (g/day), and fatty acid subtypes—saturated, monounsaturated, and polyunsaturated fats (g/day)—were calculated from the FFQ responses using a specifically designed software based on Spanish food composition tables [15]. Energy contributions from macronutrients were computed using standard conversion factors: 4 kcal/g for protein and carbohydrates, and 9 kcal/g for fats and fatty acids.

Intake of branched-chain amino acids (BCAAs)—valine, leucine, and isoleucine—was estimated from the FFQ data using the USDA amino acid composition database [16]. The individual BCAA intake in mg/d was summed and converted into grams per day (g/d). The first approach to energy adjustment considered the energetic contribution of BCAAs relative to the total energy intake: each gram of BCAA was multiplied by 4 kcal and divided by the total energy intake (kcal), yielding the percentage of energy from BCAAs (%E). This was scaled to represent increments of 0.5%E and categorized into tertiles. BCAA values were calculated for each food item reported in the baseline and 10-year FFQs and then summed. In the second approach, the BCAA values were regressed on the total energy intake (kcal/d) separately for men and women, and residuals were added to the sex-specific means, following the residual method [17]. This variable was then also scaled to represent increments of 5 g/d. Additionally, the BCAA intake adjusted for energy was divided into tertiles. Lastly, the BCAA intake was categorized by source: animal-based or plant-based (Table S1).

### 2.3. Diagnosis of Type 2 Diabetes Incidence

The identification of T2DM cases in the SUN cohort has been previously described in detail [18]. Briefly, at baseline and in each follow-up questionnaire, participants were asked whether they had been diagnosed with diabetes since the last questionnaire. Prevalent cases were defined as those who reported a previous medical diagnosis of diabetes or who were using insulin or oral antidiabetic agents at baseline. Incident cases were those participants who reported a new diagnosis of diabetes during follow-up but had not declared diabetes at baseline. To confirm the diagnosis, participants completed an additional questionnaire, including details on the type of diabetes and date of diagnosis. They were also asked to provide a medical report. All incident T2DM cases were confirmed by an independent physician blinded to the exposure. Diagnosis was based on the criteria of the American Diabetes Association: fasting plasma glucose ≥126 mg/dL (7.0 mmol/L), 2 h plasma glucose ≥200 mg/dL (11.1 mmol/L) during an oral glucose tolerance test, HbA1c ≥6.5%, or random plasma glucose ≥200 mg/dL (11.1 mmol/L) in a patient with classic symptoms of hyperglycemia or hyperglycemic crisis [19].

### 2.4. Covariate Assessment

The baseline questionnaire collected information on sociodemographic variables (e.g., marital status, educational level), validated anthropometric measurements [20], lifestyle habits (e.g., smoking status, physical activity, television viewing hours), and medical history (e.g., cancer, hypertension, hypertriglyceridemia, hypercholesterolemia, prevalent cardiovascular disease, and family history of T2DM). Physical activity was assessed using the validated Spanish version of the questionnaire from the Harvard Nurses’ Health Study and Health Professionals Follow-Up Study. Leisure-time activities were expressed in metabolic equivalents of task (METs) per week, calculated by multiplying the typical energy expenditure of each activity by the number of hours per week dedicated to it [21]. Adherence to the Mediterranean diet was assessed using the Trichopoulou score, with higher scores indicating greater adherence to this dietary pattern [22]. Missing data on television viewing (17.8%) were imputed using linear regression models, including age, sex, BMI, prevalent cardiovascular disease, and Mediterranean diet adherence (Trichopoulou score).

### 2.5. Statistical Analysis

The baseline characteristics of participants were described according to tertiles of BCAA intake, both as the percentage of total energy intake and as the energy-adjusted intake in grams per day. Continuous variables were summarized using the means and standard deviations, while categorical variables were presented as percentages. Participants were classified into tertiles based on BCAA intake expressed as a percentage of total energy intake and as energy-adjusted grams per day.

The follow-up time was defined as the period from the date of completion of the baseline questionnaire to either the date of T2DM diagnosis (as reported in follow-up questionnaires) or the date of the last follow-up questionnaire if the participant did not develop T2DM. Multivariable models were constructed based on prior knowledge of potential causal factors, as identified in the scientific literature. Analyses were stratified by recruitment period (1999–2000, 2002–2004, 2005–2007, 2008–2010, 2011–2014, ≥2015) and age categories by decade (≤30, 30–40, 50–60, 60–70, >70 years). Four models were built for multivariable adjustment: model 1 included a crude model; model 2 further adjusted for smoking status (never, current, former), cumulative tobacco exposure (pack-years), weight gain (≥5 kg or not), years of university education, television viewing (hours/day), family history of T2DM (yes/no), physical activity (MET-hours/week), adherence to the Mediterranean diet using the Trichopoulou score (low: 0–3, moderate: 4–6, high: 7–9), total energy intake (kcal/day), sugar-sweetened beverage consumption (servings/day), snacking between meals (yes/no), special diet (yes/no), prevalent hypertension (yes/no), cancer (yes/no), hypercholesterolemia (yes/no), hypertriglyceridemia (yes/no), and prevalent cardiovascular disease (yes/no); the fully adjusted model 3 was further adjusted for body mass index (BMI) and protein intake from non-BCAA sources, calculated as the sum of total amino acids minus BCAAs (g/day); and model 4 repeated the measurements after 10 years.

Cox proportional hazards regression models were used to estimate hazard ratios (HRs) and 95% confidence intervals (CIs), taking age as the underlying time variable, stratified for age (decades) and recruitment period. The lowest tertile of BCAA intake was considered the reference category. To facilitate interpretation, BCAA intake was also modeled as a continuous variable scaled to reflect increments of 0.5% of total energy and 5 g/day. Nelson–Aalen cumulative hazard estimates were computed to assess the T2DM incidence over time, stratified by the BCAA intake tertiles (% energy). These curves were adjusted using inverse probability weighting (IPW) based on the covariates included in model 3. To assess long-term dietary changes, we conducted repeated measures analyses using data from participants who completed a second FFQ 10 years after baseline (Q10 questionnaire). The average BCAA intake over time was calculated and used in the analyses. For repeated measures analyses, the cumulative average of the BCAA intake was computed to better reflect long-term habitual intake and reduce within-person variability. Specifically, for each participant, the BCAA intake from the baseline FFQ and the 10-year follow-up FFQ (Q10) was averaged. If the participant had missing data at 10 years, only the baseline intake was used. This cumulative average was then categorized into tertiles, and the median value of each tertile was assigned to all participants within that tertile. The median values were subsequently included as a continuous variable in the Cox proportional hazards models to estimate a linear *p*-trend across tertiles, reflecting the dose–response relationship over time while accounting for changes in diet during follow-up.

Stratified analyses were conducted by sex (male/female), age (<50/≥50 years), and anthropometric categories (normal weight/overweight). Interaction *p*-values were derived using likelihood ratio tests in model 3, incorporating multiplicative interaction terms between the modifying variables and BCAA intake tertiles.

The dietary sources of BCAAs were identified by food groups (Figure S1). Additional analyses were performed for the animal- and plant-based BCAA intake separately, stratified by overweight status (BMI ≥ 25 kg/m^2^) and sex. In these models, the animal BCAA intake was adjusted for the plant BCAA intake, and vice versa.

Additional sensitivity analyses excluded participants with cancer, hypertension, hypertriglyceridemia, implausible energy intake (based on Willett’s cutoffs) [17], or extreme BCAA intake (<p1 and >p99). We also conducted analyses restricted to subgroups: those with a family history of T2DM, participants with overweight (BMI ≥ 25 kg/m^2^), and sedentary individuals (physical activity <p50 in METs/week).

All *p*-values were two-sided, and statistical significance was set at *p* < 0.05. Also, the *p*-values for comparisons between BCAA tertiles were adjusted for multiple testing using the Bonferroni correction. Given the low number of incident T2DM cases and the large number of covariates included in the Cox models [23], we also conducted a permutation analysis on the main models. In this approach, the sex and BMI categories were randomly reassigned across participants to test whether the observed associations between BCAA intake and diabetes risk could have arisen by chance; empirical *p*-values were derived from 1000 permutations. All the analyses were conducted using STATA version 16.0 (StataCorp, College Station, TX, USA). A summary of the analyses is showed in Table 1.

## 3. Results

This section presents the descriptive and longitudinal analyses examining the baseline characteristics and the prospective association between dietary BCAA intake and the risk of developing type 2 diabetes. After a median follow-up of 14.67 (±5.8) years, we identified 220 incident cases of T2DM.

### 3.1. Baseline Characteristics

Table 2 shows that participants in the highest tertile of BCAA energy contribution (>3.58%) were more likely to be women (68.8% in T3) and had a higher prevalence of a family history of T2DM (17.1%). The T3 participants were older, with a slightly higher BMI, and had a higher prevalence of hypertension and cardiovascular disease. The smoking prevalence decreased across tertiles, with more never smokers in T2 and T3. Those in T3 were more likely to follow a special diet, less likely to snack, and had slightly higher physical activity levels. The energy intake decreased across tertiles, with the protein contribution increasing and carbohydrates decreasing, while the lipid intake remained stable.

### 3.2. Association Between BCAAs and Risk of Incident Diabetes

**Figure 2:**
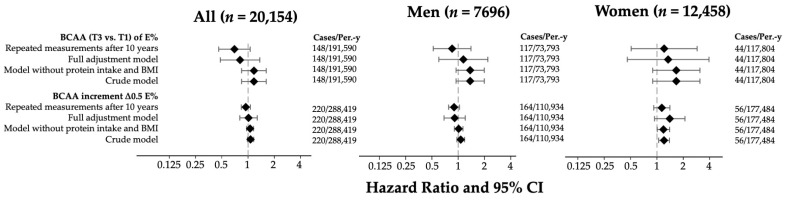
Hazard ratios and 95% CIs for tertiles (T3 vs. T1) and ∆0.5 units of BCAAs (%E) in the SUN cohort. Models adjusted for age (years), sex, smoking status (non-smokers, current, former), pack-years of smoking (continuous), weight change in ≥+5 kg (yes/no), years of university education (continuous), hours of television viewing per day (continuous), family history of T2DM, physical activity (METs-h/week), Trichopoulou score tertiles, total energy intake (kcal/d), sugary beverage consumption (g/d), snacking between meals (yes/no), special diet (yes/no), prevalent hypertension (yes/no), prevalent cancer (yes/no), prevalent hypercholesterolemia (yes/no), prevalent hypertriglyceridemia (yes/no), prevalent cardiovascular disease (yes/no), BMI (kg/m^2^), and protein intake (g/day). Abbreviations, BCAAs: branched-chain amino acids; CI: confidence interval.

The hazard ratios and 95% CIs for the risk of incident diabetes are shown in Figure 2. A total of 220 cases of incident diabetes and 288,418.66 person-years were observed during follow-up. For each 0.5% increase in energy intake from BCAAs, the adjusted model presented a hazard ratio (HR) of 1.01 (95% CI: 0.69–1.20). When we stratified by sex, the HR was 0.91 (0.69–1.20) among men and 1.40 (0.94–2.09) among women. When the BCAA intake was assessed as tertiles of the percentage of total energy intake, the fully adjusted model in the total population suggested a non-significant inverse association when we compared the extreme tertiles (HR_T3vsT1_ = 0.81; 95% CI: 0.48–1.37, *p*-trend = 0.84; Bonferroni-corrected *p* = 1.00), which became stronger after accounting for repeated dietary measures (HR_T3vsT1_ = 0.70; 95% CI: 0.46–1.06, *p*-trend = 0.06; Bonferroni-corrected *p* = 0.30). In women, both models indicated a possible positive association (HR _T3vsT1_ = 1.34; 95% CI: 0.46–3.94, Bonferroni-corrected *p* = 1.00 and HR _T3vsT1_ = 1.21; 95% CI: 0.51–2.88, Bonferroni-corrected *p* = 1.00, respectively). We also performed a permutation analysis, randomly reassigning the sex and BMI categories across participants to evaluate whether the observed associations between BCAA intake and diabetes risk could be explained by chance. The empirical *p*-values obtained were as follows: one-sided *p* = 0.56; two-sided *p* = 0.83. In men, the fully adjusted model suggested a non-significant positive association (HR _T3vsT1_ = 1.15; 95% CI: 0.60–2.19, Bonferroni-corrected *p* = 1.00), whereas the repeated measures analysis showed an inverse trend (HR _T3vsT1_ = 0.85; 95% CI: 0.52–1.40, *p*-trend = 0.04; Bonferroni-corrected *p* = 0.20). Other analyses (residual-energy-adjusted-based models) did not yield significant associations and substantially different results (Table S2).

### 3.3. Exploratory Stratified Analysis by Overweight, Sex and Age Subgroups

In stratified analyses using the BCAA intake as the energy percentage (Table 3), no statistically significant associations were observed across subgroups, including when we corrected with the Bonferroni method (Bonferroni-corrected *p* for all the strata analysis = 1.00). Among women, the risk estimates were elevated in the highest tertile (HR = 1.34; 95% CI: 0.46–3.94), whereas in men, the estimate was more modest (HR = 1.15; 95% CI: 0.60–2.19). Stratification by overweight and age showed no consistent patterns (HRs ranging 0.33–1.63), with no statistically significant interactions. When we performed an analysis that included residual-energy-adjusted tertiles, the results did not change substantially and no significant results were found.

### 3.4. Exploratory Analyses of BCAA Sources and Sensitivity Analyses

When stratifying the BCAA intake by animal versus plant sources and by sex and weight status, no statistically significant associations were observed in the overall population (highest vs. lowest tertile: HR = 1.17; 95% CI: 0.84–1.62; *p*-trend = 0.51). The exploratory patterns suggested a higher risk in obese women (total BCAA HR = 2.56; 95% CI: 0.72–9.13) and in normal-weight men for plant-derived BCAAs (HR = 2.81; 95% CI: 0.60–13.14), while normal-weight women showed a non-significant inverse trend (HR = 0.30; 95% CI: 0.08–1.22). After applying Bonferroni correction for multiple testing, none of these associations reached statistical significance, supporting the exploratory nature of these analyses. Estimates in other subgroups were closer to null.

**Figure 3:**
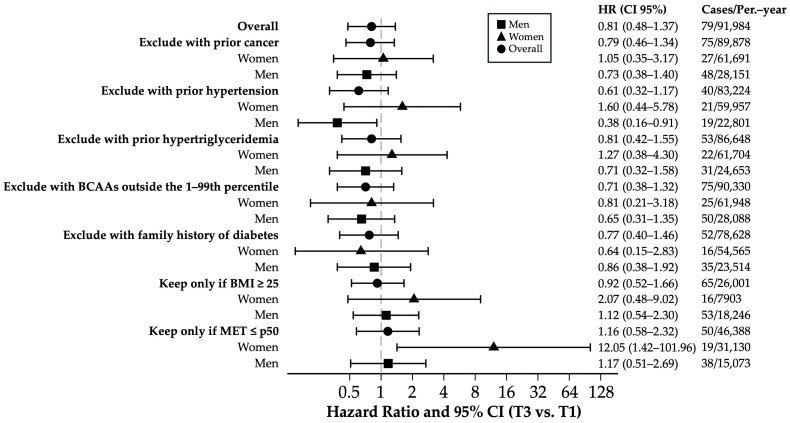
Sensitivity analysis of multivariate hazard ratios and 95% confidence intervals comparing T3 vs. T1 for incident T2DM across tertiles of the percentage of caloric intake of BCAAs. Hazard ratio and 95% CI of T3 vs. T1 adjusted for age (years), sex, smoking status (non-smokers, current, former), pack-years of smoking (continuous), weight change in ≥+5 kg (yes/no), years of university education (continuous), hours of television viewing per day (continuous), family history of T2DM, physical activity (METs-h/week), Trichopoulou score tertiles, total energy intake (kcal/d), sugary beverage consumption (g/d), snacking between meals (yes/no), special diet (yes/no), prevalent hypertension (yes/no), prevalent cancer (yes/no), prevalent hypercholesterolemia (yes/no), prevalent hypertriglyceridemia (yes/no), prevalent cardiovascular disease (yes/no), and BMI (kg/m^2^). Abbreviations, HR: hazard ratio; T2DM: type 2 diabetes mellitus; BCAAs: branched-chain amino acids; HBP: arterial hypertension; BMI: body mass index; METs: metabolic equivalents of activity.

Sensitivity analyses (Figure 3) comparing the highest vs. lowest tertile of BCAA energy intake showed no significant association with the T2DM risk in the overall population (HR = 0.81; 95% CI = 0.48–1.37; *p* = 0.43, Bonferroni-corrected *p* = 1.00). The results were consistent after excluding participants with prior cancer, hypertension, hypertriglyceridemia, extreme BCAA values, or a family history of diabetes (HRs = 0.61–0.81; corrected *p*-values = 1.00), although these estimates are limited by the low number of incident cases and wide confidence intervals. The sex-stratified analyses were generally non-significant; among men without prior hypertension, the HR suggested a potentially protective trend (HR = 0.38; 95% CI = 0.16–0.91; *p* = 0.029, corrected *p* = 0.64), but the low number of cases reduces the statistical power and makes this finding exploratory. In sedentary women (MET ≤ p50), a high HR was observed (HR = 12.05; 95% CI = 1.42–101.96; *p* = 0.022, corrected *p* = 0.49), yet the wide confidence interval reflects the limited number of events, indicating this pattern is suggestive but not conclusive. Other estimates in subgroups similarly showed wide confidence intervals, implying limited precision due to low incidence, and should be interpreted cautiously.

## 4. Discussion

In our study, we observed that overall dietary BCAA intake was not significantly associated with incident T2DM in the total population. Moreover, the empirical *p*-values from the permutation analysis (0.56 one-sided and 0.83 two-sided) indicated that the observed estimates were not more extreme than expected under the null hypothesis, further supporting the notion that the associations could be explained by chance rather than representing a robust effect. When using repeated dietary measurements, a modest inverse trend was suggested, whereas in women, the estimates indicated a possible positive association, and in men, the associations were inconsistent, showing both non-significant positive and inverse trends. Analyses by BCAA source (animal vs. plant) revealed heterogeneous patterns without statistical significance, although exploratory signals suggested a higher risk among overweight women consuming more animal-derived BCAAs and some inverse trends in men. Across all the analyses, the confidence intervals were wide, reflecting the low number of incident T2DM cases and limited statistical power, which underscores the exploratory nature of these findings.

To our knowledge, only three longitudinal studies have examined the association between BCAA intake and T2DM risk in Japanese, American, and Iranian populations [24,25,26]. This is the first prospective study in a Spanish cohort to evaluate both the total BCAA intake and the intake by animal or plant origin in relation to incident T2DM. Comparisons with prior studies show both consistencies and discrepancies, reflecting differences in population characteristics, dietary patterns, BCAA assessment methods, and outcome definitions. While the general trend in our study aligns with some previous reports suggesting higher BCAA intake may be linked to the T2DM risk, particularly among women, the magnitude and direction of associations varied across studies, highlighting the preliminary and exploratory character of our findings.

In our cohort, higher BCAA intake in women was associated with increased risk of developing T2DM independently of multiple diabetes risk factors, including BMI. Higher intake of animal-derived BCAAs also appeared to be associated with increased T2DM risk in women with overweight. When the BCAA intake was categorized in tertiles, the associations were not statistically significant, potentially due to the small differences in mean intake across tertiles, leading to a homogeneous distribution of cases and possibly masking associations in the categorical analyses. The linear models, however, revealed trends that support potential associations, suggesting that BCAA intake may contribute to T2DM risk in certain subgroups.

Comparisons with previous studies illustrate the variability of the findings. The Iranian study reported a positive association between BCAA intake and insulin resistance, while the Japanese study observed an inverse association in women and no association in men [25,26]. The U.S. studies showed increased T2DM risk with higher BCAA intake in women and men, although methodological differences such as energy adjustment, population age and BMI, and BCAA source proportions may contribute to the discrepancies [24]. Our study adds to this literature by providing exploratory evidence in a Mediterranean population, with dietary sources of BCAAs comprising approximately 50% meat, eggs, and fish, 25% dairy, and 25% plant sources.

As strengths of this study, the prospective design clarifies the time sequence while avoiding bias. The study has a long follow-up period (13 years on average) and a retention rate of over 90%, which partially avoids selection bias. Incident cases of T2DM are confirmed by a blinded physician and are self-reported. Dietary information is collected using the validated FFQ, and we have repeated measures of the total caloric intake and BCAAs at 10 years. Furthermore, the highly educated cohort increases the reliability of self-reported information, which strengthens the internal validity and controls for confounding due to socioeconomic factors. The multivariate adjustment model controls for environmental and lifestyle confounding. The main limitations are the low number of incident T2DM cases, which limits the statistical power of the study and may have prevented detection of additional associations. Given that our observed number of events is lower than the number required to reach conventional 80% power for many of the hazard ratios observed, this limitation is explicitly acknowledged in this study. Consequently, all the subgroup and repeated measures analyses are considered exploratory. On the other hand, we cannot exclude the possibility of measurement errors in self-reported variables such as BCAA calculations, total energy intake, or many other covariates in the adjustment model. However, as previously mentioned, the reliability of the information is high due to the participants’ high educational level. We were unable to control for hormonal or genetic factors, which affects the study’s internal validity. We also cannot exclude the possibility of residual confounding due to the lack of a randomized design. Finally, external validity could be compromised by the fact that only university graduates participated.

## 5. Conclusions

This exploratory study examined the potential associations between dietary BCAA intake and T2DM risk. While some suggestive patterns were observed, particularly in women, most associations were not statistically significant and should be interpreted cautiously due to the limited number of incident cases and wide confidence intervals. Analyses by BCAA source also showed non-significant and heterogeneous patterns, highlighting the exploratory nature of these findings. Given the low statistical power of the study, these results do not allow for definitive conclusions. Future studies should include larger and more diverse populations to ensure sufficient events for reliable estimation and to better evaluate the potential associations between dietary BCAAs and the T2DM risk.

## Figures and Tables

**Table 1:** Summary of the analyses.

Analysis Type	Description	Purpose	Models/Adjustments	Multiple Testing/Validation
Primary analysis	Cox regression by tertiles of baseline BCAA intake (% energy and g/day)	Main test of association with incident T2DM	Models 1–3 (crude, lifestyle/clinical covariates, +BMI and non-BCAA protein)	Bonferroni correction across tertiles; formal power calculation included
Permutation analysis	Random reassignment of sex and BMI across participants (1000 permutations)	Test whether observed associations could arise by chance	Main models	Empirical *p*-values reported
Dose–response (trend test)	Median of tertiles modeled as continuous (linear *p*-trend)	Assess trend across tertiles	Same as primary analysis	Bonferroni correction
Repeated measures analysis	Cumulative average of baseline and 10 y FFQ BCAA intake, categorized in tertiles	Capture long-term habitual intake	Models 1–4 (incl. repeated measures)	Linear *p*-trend across tertiles
Stratified analyses	By sex, age (<50/≥50), BMI categories (normal/overweight)	Explore effect modification	Model 3 + interaction terms	Exploratory, no correction
Sources of BCAAs	Animal- vs. plant-derived BCAA intake (mutually adjusted)	Assess heterogeneity by source	Model 3	Exploratory, no correction
Sensitivity analyses	Excluding participants with cancer, hypertension, hypertriglyceridemia, implausible energy intake, extreme BCAA values	Robustness of findings	Model 3	Not adjusted (secondary)
Restricted subgroup analyses	Family history of T2DM, overweight, sedentary participants	Check consistency in specific groups	Model 3	Exploratory, no correction

**Table 2:** Baseline characteristics according to tertiles of energy intake from BCAAs in the SUN cohort.

	BCAAs Intake (%E)			
	T1 (<3)	T2 (3 to 3.58)	T3 (>3.58)	*p*
N	6718	6718	6718	
BCAAs (g/day)	18.5 (5.5)	20.4 (5.7)	22.6 (7.4)	<0.001
BCAAs (%E*)	2.6 (0.3)	3.3 (0.2)	4.1 (0.5)	<0.001
Isoleucine (g/d)	4.7 (1.4)	5.2 (1.5)	5.8 (1.9)	<0.001
Leucine (g/d)	8.3 (2.5)	9.2 (2.6)	10.1 (3.4)	<0.001
Valine (g/d)	5.5 (1.6)	6.0 (1.7)	6.7 (2.2)	<0.001
Women (%)	54.2%	62.4%	68.8%	<0.001
Age (y)	37.9 (12.0)	36.8 (11.8)	38.2 (12.4)	<0.001
BMI (kg/m^2^)	23.3 (3.5)	23.2 (3.4)	23.7 (3.6)	<0.001
Smoking (pack-years)	6.1 (9.8)	5.0 (8.7)	5.8 (9.7)	<0.001
Smoking Habit				<0.001
Never smokers (%)	47.2%	50.9%	48.6%	
Smokers (%)	27.1%	25.1%	24.5%	
Ex-smokers (%)	24.9%	23.3%	26.2%	
Missing values (%)	0.7%	0.7%	0.7%	
Marital Status				<0.001
Single (%)	46.1%	47.2%	43.2%	
Married (%)	48.8%	47.9%	50.8%	
Other (%)	5.1%	5.0%	6.0%	
College education (years)	5.1 (1.5)	5.0 (1.5)	5.0 (1.5)	0.003
Physical activity (METs-h/week)	21.5 (22.4)	22.1 (23.2)	22.7 (24.3)	0.009
Television viewing (h/d)	1.6 (1.2)	1.6 (1.2)	1.6 (1.1)	0.647
Family history of T2DM (%)	13.7%	14.8%	17.0%	<0.001
Snacking between meals (%)	37.0%	34.7%	32.0%	<0.001
Weight change (≥+5 kg) ^1^ (%)	12.7%	11.8%	13.0%	0.078
Special diet (%)	5.1%	5.5%	12.0%	<0.001
Prevalence of CVD (%)	1.4%	1.0%	1.7%	0.003
Prevalence of HBP (%)	10.4%	8.8%	11.4%	<0.001
Prevalence of cancer (%)	2.6%	2.2%	2.7%	0.174
Prevalence of hypercholesterolemia (%)	15.9%	15.3%	18.0%	<0.001
Prevalence of hypertriglyceridemia (%)	5.9%	6.1%	6.8%	0.077
Trichopoulou Mediterranean Diet Scale				0.023
0–3 points (%)	34.5%	36.3%	35.9%	
4–5 points (%)	39.0%	38.7%	39.8%	
6–9 points (%)	26.5%	25.0%	24.3%	
Diet				
Energy intake (kcal/d)	2802 (816)	2492 (698)	2197 (686)	<0.001
Carbohydrates (%E*)	46.6 (7.3)	43.7 (6.5)	40.6 (7.4)	<0.001
Proteins (%E*)	14.7 (1.7)	18.1 (0.9)	22.5 (2.8)	<0.001
Lipids (%E*)	36.1 (6.9)	36.6 (6.2)	36.3 (6.7)	<0.001
Monounsaturated fatty acids (%E*)	15.9 (4.0)	15.9 (3.5)	15.5 (3.5)	<0.001
Polyunsaturated fatty acids (%E*)	5.7 (1.8)	5.2 (1.4)	4.8 (1.3)	<0.001
Saturated fatty acids (%E*)	12.0 (3.1)	12.7 (3.0)	12.9 (3.6)	<0.001
Glycemic load	179.0 (65.7)	144.3 (50.0)	111.8 (44.3)	<0.001
Dairy products (g/day)	226.5 (207.9)	219.6 (217.1)	179.7 (217.6)	<0.001
Red meat (g/day)	71.6 (43.8)	83.6 (46.7)	84.2 (58.2)	<0.001
Vegetables (g/day)	502.7 (350.6)	544.2 (351.0)	602.3 (392.9)	<0.001
Fruit (g/day)	319.3 (333.6)	304.6 (266.6)	292.3 (242.7)	<0.001
Fish (g/day)	76.1 (44.2)	98.2 (50.8)	130.2 (87.5)	<0.001
Legumes (g/day)	24.8 (23.1)	24.2 (19.6)	22.3 (16.9)	<0.001
Cereals (g/day)	134.5 (95.7)	111.3 (74.2)	81.8 (59.4)	<0.001
Fiber (g/day)	26.5 (13.0)	23.9 (10.7)	21.6 (9.9)	<0.001
Olive oil (g/day)	23.6 (18.8)	19.2 (14.5)	15.4 (12.0)	<0.001
Coffee (g/day)	64.2 (66.2)	59.0 (60.9)	59.5 (64.6)	<0.001
Sweetened beverages (servings/day)	60.0 (120.1)	40.3 (69.2)	28.3 (63.2)	<0.001

Abbreviations: BCAAs: branched-chain amino acids; METs: metabolic equivalents of protein; DM: diabetes mellitus; CVD: cardiovascular disease; HBP: hypertension; BMI: body mass index. %E*: percentage of caloric intake. ^1^ Gaining 5 kg or more of weight, or not during the last 5 years before baseline.

**Table 3:** Exploratory stratified analysis for tertiles of energy intake from BCAAs by age, sex, and overweight subgroups.

			HR (IC 95%)			
Model	*n*	Cases/Person-Years	T1	T2	T3	*p*-Interaction
BCAA (%E)						
Overweight	5827	181/80,793	1 (Ref.)	1.14 (0.74–1.77)	0.93 (0.52–1.66)	0.521
Normoweight	14,327	39/207,626	1 (Ref.)	0.55 (0.22–1.41)	0.33 (0.08–1.34)	
Women	12,458	56/177,484	1 (Ref.)	0.88 (0.36–2.15)	1.34 (0.46–3.94)	0.308
Men	7696	164/110,934	1 (Ref.)	1.09 (0.67–1.77)	1.15 (0.60–2.19)	
Age ≥ 50 years	3459	131/47,026	1 (Ref.)	0.88 (0.54–1.44)	0.65 (0.33–1.27)	0.741
Age < 50 years	16,695	89/241,393	1 (Ref.)	1.56 (0.79–3.06)	1.63 (0.66–4.03)	

Models were stratified by the variable of interest and did not include it as a covariate. All models were adjusted for age (years), sex, smoking status (non-smokers, current, former), pack-years of smoking (continuous), weight change in ≥+5 kg (yes/no), years of university education (continuous), hours of television viewing per day (continuous), family history of T2DM, physical activity (METs-h/week), Trichopoulou score tertiles, total energy intake (kcal/d), sugary beverage consumption (g/d), snacking between meals (yes/no), special diet (yes/no), prevalent hypertension (yes/no), prevalent cancer (yes/no), prevalent hypercholesterolemia (yes/no), prevalent hypertriglyceridemia (yes/no), prevalent cardiovascular disease (yes/no), BMI (kg/m^2^), and protein intake (g/day). Abbreviations, HR: Hazard Ratio.

## Data Availability

This study uses data from the Seguimiento Universidad de Navarra (SUN) cohort. All data and materials, as well as software application or custom code, used during the current study shall be made available by the corresponding author on reasonable request.

## Supplementary Materials

The following supporting information can be downloaded at https://www.mdpi.com/article/10.3390/biomedicines13102561/s1. Table S1: Animal-based and plant-based BCAA in SUN cohort; Figure S1: BCAA sources in SUN cohort; Table S2: Hazard Ratios and 95% CI for a 0.5% increase in total energy intake, tertiles of energy intake from BCAAs, 5 g/day increments and residual-based tertiles of BCAA intake in the SUN cohort.

## Abbreviations

The following abbreviations are used in this manuscript:

ADA	American Diabetes Association
BCAA(s)	Branched-Chain Amino Acid(s)
BMI	Body Mass Index (Índice de Masa Corporal, implícito)
CVD	Cardiovascular Disease
FFQ	Food Frequency Questionnaire
HbA1c	Hemoglobin A1c
HPFS	Health Professionals Follow-up Study
HR	Hazard Ratio
IR	Insulin Resistance
MET(s)	Metabolic Equivalent(s) of Task
mTOR	Mechanistic Target of Rapamycin
NHS	Nurses’ Health Study
NHS II	Nurses’ Health Study II
SUN	Seguimiento Universidad de Navarra
T2DM	Type 2 Diabetes Mellitus
USDA	United States Department of Agriculture
%E	Percentage of Total Energy Intake from BCAAs

## References

[1] Arroyo-Cerezo A., Cerrillo I., Ortega Á., Fernández-Pachón M.-S. (2021). Intake of Branched Chain Amino Acids Favors Post-Exercise Muscle Recovery and May Improve Muscle Function: Optimal Dosage Regimens and Consumption Conditions. J. Sports Med. Phys. Fit..

[2] Martinho D.V., Nobari H., Faria A., Field A., Duarte D., Sarmento H. (2022). Oral Branched-Chain Amino Acids Supplementation in Athletes: A Systematic Review. Nutrients.

[3] Matsuda T., Suzuki H., Sugano Y., Suzuki Y., Yamanaka D., Araki R., Yahagi N., Sekiya M., Kawakami Y., Osaki Y. (2022). Effects of Branched-Chain Amino Acids on Skeletal Muscle, Glycemic Control, and Neuropsychological Performance in Elderly Persons with Type 2 Diabetes Mellitus: An Exploratory Randomized Controlled Trial. Nutrients.

[4] McCormack S.E., Shaham O., McCarthy M.A., Deik A.A., Wang T.J., Gerszten R.E., Clish C.B., Mootha V.K., Grinspoon S.K., Fleischman A. (2013). Circulating Branched-chain Amino Acid Concentrations Are Associated with Obesity and Future Insulin Resistance in Children and Adolescents. Pediatr. Obes..

[5] Zhao X., Han Q., Liu Y., Sun C., Gang X., Wang G. (2016). The Relationship between Branched-Chain Amino Acid Related Metabolomic Signature and Insulin Resistance: A Systematic Review. J. Diabetes Res..

[6] Guasch-Ferré M., Hruby A., Toledo E., Clish C.B., Martínez-González M.A., Salas-Salvadó J., Hu F.B. (2016). Metabolomics in Prediabetes and Diabetes: A Systematic Review and Meta-Analysis. Diabetes Care.

[7] van Nielen M., Feskens E.J.M., Mensink M., Sluijs I., Molina E., Amiano P., Ardanaz E., Balkau B., Beulens J.W.J., Boeing H. (2014). Dietary Protein Intake and Incidence of Type 2 Diabetes in Europe: The EPIC-InterAct Case-Cohort Study. Diabetes Care.

[8] Mensink M. (2024). Dietary Protein, Amino Acids and Type 2 Diabetes Mellitus: A Short Review. Front. Nutr..

[9] Vieira E.E.S., Pereira I.C., Braz A.F., Nascimento-Ferreira M.V., de Oliveira Torres L.R., de Freitas Brito A., do Nascimento Marreiro D., de Castro e Sousa J.M., da Silva F.C.C., Torres-Leal F.L. (2020). Food Consumption of Branched Chain Amino Acids and Insulin Resistance: A Systematic Review of Observational Studies in Humans. Clin. Nutr. ESPEN.

[10] Ramzan I., Ardavani A., Vanweert F., Mellett A., Atherton P.J., Idris I. (2022). The Association between Circulating Branched Chain Amino Acids and the Temporal Risk of Developing Type 2 Diabetes Mellitus: A Systematic Review & Meta-Analysis. Nutrients.

[11] Ancu O., Mickute M., Guess N.D., Hurren N.M., Burd N.A., Mackenzie R.W. (2021). Does High Dietary Protein Intake Contribute to the Increased Risk of Developing Prediabetes and Type 2 Diabetes?. Appl. Physiol. Nutr. Metab..

[12] White P.J., McGarrah R.W., Herman M.A., Bain J.R., Shah S.H., Newgard C.B. (2021). Insulin Action, Type 2 Diabetes, and Branched-Chain Amino Acids: A Two-Way Street. Mol. Metab..

[13] Wang S., Li Y., Wang M., Yuan J., Zeleznik O.A., Eliassen A.H., Chan A.T., Hu F.B., Hu Y., Sun Q. (2025). Amino Acid Intake, Plasma Metabolites, and Incident Type 2 Diabetes Risk: A Systematic Approach in Prospective Cohort Studies. Nutr. J..

[14] Martínez-González M.Á. (2006). The SUN Cohort Study (Seguimiento University of Navarra). Public Health Nutr..

[15] Moreiras O., Tuni O.M. (2009). Tablas de Composición de Alimentos: Guía de Prácticas.

[16] de la O V., Zazpe I., de la Fuente-Arrillaga C., Santiago S., Goni L., Martínez-González M.Á., Ruiz-Canela M. (2023). Association between a New Dietary Protein Quality Index and Micronutrient Intake Adequacy: A Cross-Sectional Study in a Young Adult Spanish Mediterranean Cohort. Eur. J. Nutr..

[17] Willett W.C. (2013). Nutritional Epidemiology.

[18] Martínez-González M.Á., de la Fuente-Arrillaga C., Nunez-Cordoba J.M., Basterra-Gortari F.J., Beunza J.J., Vazquez Z., Benito S., Tortosa A., Bes-Rastrollo M. (2008). Adherence to Mediterranean Diet and Risk of Developing Diabetes: Prospective Cohort Study. BMJ.

[19] Valer-Martinez A., Sayon-Orea C., Martinez J.A., Basterra-Gortari F.J., Martinez-Gonzalez M.A., Bes-Rastrollo M. (2024). Vitamin D and Risk of Developing Type 2 Diabetes in the SUN Project: A Prospective Cohort Study. J. Endocrinol. Investig..

[20] Bes-Rastrollo M., Valdivieso J.R., Sanchez-Villegas A., Alonso A., Martínez-González M. (2005). Validation of Self-Reported Weight and Body Mass Index of the Participants of a Cohort of University Graduates. Rev. Esp. Obes..

[21] Martínez-González M.A., López-Fontana C., Varo J.J., Sánchez-Villegas A., Martinez J.A. (2005). Validation of the Spanish Version of the Physical Activity Questionnaire Used in the Nurses’ Health Study and the Health Professionals’ Follow-up Study. Public Health Nutr..

[22] Trichopoulou A., Costacou T., Bamia C., Trichopoulos D. (2003). Adherence to a Mediterranean Diet and Survival in a Greek Population. N. Engl. J. Med..

[23] Holt C.A., Sullivan S.P. (2023). Permutation Tests for Experimental Data. Exp. Econ..

[24] Zheng Y., Li Y., Qi Q., Hruby A., Manson J.E., Willett W.C., Wolpin B.M., Hu F.B., Qi L. (2016). Cumulative Consumption of Branched-Chain Amino Acids and Incidence of Type 2 Diabetes. Int. J. Epidemiol..

[25] Nagata C., Nakamura K., Wada K., Tsuji M., Tamai Y., Kawachi T. (2013). Branched-Chain Amino Acid Intake and the Risk of Diabetes in a Japanese Community: The Takayama Study. Am. J. Epidemiol..

[26] Asghari G., Farhadnejad H., Teymoori F., Mirmiran P., Tohidi M., Azizi F. (2018). High Dietary Intake of Branched-Chain Amino Acids Is Associated with an Increased Risk of Insulin Resistance in Adults. J. Diabetes.

