# Supplementary material for: Branched-Chain Amino Acid Intake and Risk of Incident Type 2 Diabetes: Results from the SUN Cohort
Source: Biomedicines. 2025 Oct 21;13(10):2561. doi: 10.3390/biomedicines13102561 (PMC12561068; doi:10.3390/biomedicines13102561)
Supplement: Supplementary file 1 [file biomedicines-13-02561-s001.zip › biomedicines-3819321-supplementary.pdf]

Table S1. Animal-based and plant-based BCAA in SUN cohort.

VEGETAL	ANIMAL
Chard and spinach (250g serving)	Whole milk consumption: 1 cup: 200 ml
Cabbage, cauliflower, and broccoli (250g serving)	Semi-skimmed milk consumption: 1 cup: 200 ml
Lettuce, endives, and escarole (250g serving)	Skimmed milk consumption: 1 cup: 200 ml
Raw tomato (150g serving)	Condensed milk consumption: 1 tablespoon
Carrots and squash (250g serving)	Cream or heavy cream consumption: 1/2 cup
Green beans (250g serving)	Milkshake consumption: 1 cup: 200 ml
Eggplant, zucchini, and cucumber (250g serving)	Whole yogurt consumption: 1 unit, 125 grams
Peppers (250g serving)	Skimmed yogurt consumption: 1 unit, 125 grams
Asparagus (250g serving)	Petit Suisse consumption: 1 unit, 100 grams
Gazpacho (250g serving)	Cottage or curd consumption: 1/2 cup
Other vegetables (borage, cardoon, etc.) (250g serving)	Portioned or cream cheese consumption: 1 portion
Homemade potato chips (bag, 150g serving)	Other cheeses (Manchego, round, Emmental... 50 g)
Baked or boiled potatoes (150g serving) 150g	White or fresh Burgos cheese, goat cheese... 50 g
Orange, grapefruit (one), or mandarin (two)	Custard, flan, pudding consumption: 1 cup, 200cc
Banana (one)	Ice cream consumption per unit
Apple, pear (one)	Hen's eggs consumption per unit
Strawberries (6), dessert plate	Chicken or turkey consumption (with skin)
Peach, apricot, nectarine (one)	Chicken or turkey consumption (without skin)
Cherries, picotas, plums (one dessert plate)	Veal or beef consumption (with pork)
Figs (one)	Lamb consumption (with lamb)
Watermelon (1 slice), 200-250g	Rabbit meat consumption (with liver)
Melon (1 slice), 200-250g	Other organ meats consumption: brains, heart, sweetbreads, etc.
Grapes (one bunch), dessert plate	Serrano ham or shoulder ham consumption (with shoulder ham)
Fruit in syrup (2)	York ham consumption (with 1 slice)
Fruit in juice (2)	Cold cuts consumption: chorizo, salami, mortadella, etc. (50g)
Dates, dried figs, raisins, prunes (150g)	Sausage consumption (with 50g)
Nuts: almonds, peanuts, hazelnuts n	Pâté, foie gras consumption (with 25g)
Olives: 10 units	Black pudding consumption (with 50g)
Avocado: 1 unit	Hamburger consumption (with ham) (with sobrasada) (with 50g) / meatballs consumption (with 3 pieces)
Mango: 1 unit	Bacon, bacon, pancetta consumption (with 50g)
Kiwi: 1 unit	White fish: whiting, hake, sea bream, grouper
Lentils: 60g dry	Bluefish consumption: sardines, tuna, bonito, mackerel, saltfish
Chickpeas: 60g dry	Cod consumption: 1 plate, piece, or portion
Beans: pinto, white, or black: 60g dry	Salted and/or smoked fish consumption: herring, salmon
Peas: 60g dry	Oysters, clams, mussels, etc. consumption: 6 units
White bread: 3 slices, 60g	Shrimp, prawns, crayfish consumption: 100-150g
Wholemeal bread: 3 slices, 60g	Octopus, squid, baby squid, cuttlefish... consumption: 100-150g
Cereals: 30g dry	Butter consumption: 1 individual portion
Rice: 60g dry	Lard consumption: 1 individual portion
Pasta: noodles, macaroni, spaghetti: 60g dry	Other oils and fats consumption: 1 tablespoon
Pizza: 1 serving: 200g	How often do you eat fried foods at home?
Margarine: 1 single serving	How often do you eat fried foods outside the home?
Olive oil: 1 tablespoon	Do you use butter for frying at home?

Sunflower oil: 1 tablespoon	Do you use other fats for frying at home?
Corn oil: 1 tablespoon	Mayonnaise consumption: 1 teaspoon
Do you use olive oil for frying?)	Honey consumption: 1 teaspoon
Do you use sunflower oil for frying?)	
Do you use corn oil for frying?)	
Do you use margarine for frying?)	
Maria cookies 4-6 units, 50g	
Chocolate cookies 4-6 units, 50g	
Commercial muffins 1-2 units	
Donuts 1	
Industrial pastries 1, 50g	
Homemade pastries 1	
Cakes 1, 50g	
Churros, porras, and similar (serving, 100g)	
Chocolate and bonbons 30g	
Nougat 90g	
Tea biscuits, mantecados, marzipan (serving, 90g)	
One glass of red wine 1 glass of other types of wine wine)	
A glass of wine with meals)	
Beer consumption: 1 jug, 330cc	
Liqueur consumption: whiskey, gin, cognac, anise...	
Sugar-sweetened carbonated beverages: coca-cola, orange juice	
Light carbonated beverages consumption: 1 small bottle, 200cc	
Natural orange juice consumption: 1 glass, 200cc	
Natural other fruit juice consumption: 1 glass, 200cc	
Canned fruit or vegetable juice consumption: 200cc	
Decaffeinated coffee consumption: 1 cup, 50cc	
Coffee consumption: 1 cup, 50cc	
Tap water consumption: 1 glass, 200cc	
Bottled water consumption: 1 glass, 200cc	
Croquettes, bunyuelos, and (empanadillas)	
Soups and creams from sachets)	
Tomato sauce, ketchup 1 teaspoon	
Spicy foods: tabasco, pepper 1 teaspoon	
Salt 1 pinch	
Sugar 1 teaspoon	
Saccharin)	
Jam 1 teaspoon	

Figure S1. BCAA sources in SUN cohort.

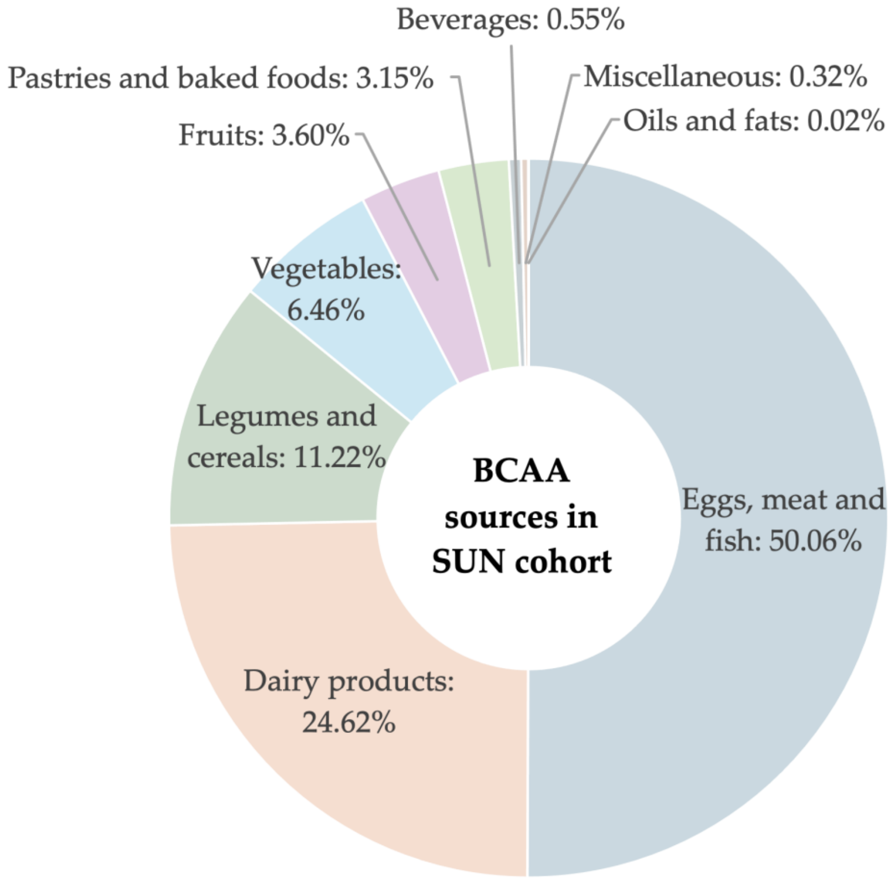

Table S2. Hazard Ratios and 95% CI for a 0.5% increase in total energy intake, tertiles of energy intake from BCAAs, 5 g/day increments and residual-based tertiles of BCAA intake in the SUN cohort.

	N	Cases*	Persons-Years	HR (IC 95%)[1]	HR (IC 95%)[2]	HR (IC 95%)[3]	HR (IC 95%)[4]	HR (IC 95%)[5]
				**0.5% increase in energy intake from BCAAs**				
**Total**	20154	220	288419	1.11 (1.02-1.21)	1.06 (0.96-1.16)	1.02 (0.93-1.11)	1.01 (0.81-1.27)	0.94 (0.84-1.06)
**Men**	7696	164	110934	1.07 (0.96-1.18)	1.01 (0.90-1.13)	0.96 (0.86-1.08)	0.91 (0.69-1.20)	0.92 (0.69-1.22)
**Women**	12458	56	177484	1.21 (1.05-1.39)	1.19 (1.01-1.40)	1.26 (1.07-1.49)	1.40 (0.94-2.09)	1.18 (0.83-1.68)
				**Tertiles % total energy from BCAAs**				
**Total**								
T1	20154	69	99606	1 (ref.)	1 (ref.)	1 (ref.)	1 (ref.)	1 (ref.)
T2	20154	72	96828	1.27 (0.91-1.76)	1.23 (0.88-1.74)	1.25 (0.88-1.75)	1.15 (0.78-1.70)	1.06 (0.74-1.51)
T3	20154	79	91984	1.33 (0.96-1.85)	1.10 (0.77-1.57)	0.95 (0.67-1.36)	0.81 (0.48-1.37)	0.70 (0.46-1.06)
			p-trend	0.09	0.67	0.68	0.34	0.06
**Men**								
T1	7696	48	38414	1 (ref.)	1 (ref.)	1 (ref.)	1 (ref.)	1 (ref.)
T2	7696	47	37142	1.06 (0.71-1.58)	1.02 (0.67-1.55)	1.01 (0.67-1.53)	1.09 (0.67-1.77)	0.91 (0.59-1.41)
T3	7696	69	35379	1.36 (0.94-1.97)	1.16 (0.77-1.74)	0.98 (0.65-1.48)	1.15 (0.60-2.19)	0.85 (0.52-1.40)
			p-trend	0.09	0.44	0.91	0.69	0.04
**Women**								
T1	12458	16	61272	1 (ref.)	1 (ref.)	1 (ref.)	1 (ref.)	1 (ref.)
T2	12458	12	59680	0.82 (0.39-1.74)	0.97 (0.45-2.12)	1.09 (0.49-2.46)	0.88 (0.36-2.15)	0.84 (0.37-1.92)
T3	12458	28	56532	1.67 (0.90-3.11)	1.70 (0.83-3.46)	2.04 (0.96-4.32)	1.34 (0.46-3.94)	1.21 (0.51-2.88)
			p-trend	0.07	0.11	0.05	0.51	0.80
				**5g increase in BCAAs adjusted for energy**				
Total	20154	220	288419	1.13 (0.99-1.30)	1.07 (0.93-1.23)	1.02 (0.88-1.18)	0.81 (0.20-3.37)	0.96 (0.77-1.20)
Men	7696	164	110934	1.07 (0.90-1.26)	1.02 (0.85-1.23)	0.95 (0.79-1.14)	0.48 (0.08-2.76)	0.92 (0.69-1.22)
Women	12458	56	177484	1.31 (1.04-1.66)	1.21 (0.95-1.54)	1.34 (1.05-1.72)	1.76 (0.14-21.47)	1.18 (0.83-1.68)
				**Tertiles of energy-adjusted BCAA intake**				
**Total**								
T1	20154	70	99478	1 (ref.)	1 (ref.)	1 (ref.)	1 (ref.)	1 (ref.)
T2	20154	76	96531	1.21 (0.88-1.68)	1.16 (0.83-1.61)	1.16 (0.83-1.62)	1.08 (0.75-1.56)	1.10 (0.78-1.56)
T3	20154	74	92410	1.24 (0.89-1.72)	1.11 (0.79-1.56)	0.95 (0.68-1.34)	0.81 (0.50-1.33)	0.90 (0.60-1.34)
			p-trend	0.22	0.56	0.73	0.39	0.57
**Men**								
T1	7696	49	38365	1 (ref.)	1 (ref.)	1 (ref.)	1 (ref.)	1 (ref.)
T2	7696	53	37123	1.16 (0.79-1.72)	1.13 (0.76-1.69)	1.14 (0.77-1.70)	1.26 (0.79-2.00)	1.17 (0.77-1.78)
T3	7696	62	35446	1.36 (0.93-1.98)	1.22 (0.83-1.80)	1.02 (0.69-1.51)	1.25 (0.66-2.39)	1.05 (0.64-1.70)
			p-trend	0.11	0.31	0.98	0.53	0.47
**Women**								
T1	12458	16	61154	1 (ref.)	1 (ref.)	1 (ref.)	1 (ref.)	1 (ref.)
T2	12458	17	59617	1.16 (0.58-2.30)	1.25 (0.61-2.58)	1.42 (0.68-2.99)	1.08 (0.49-2.36)	1.07 (0.49-2.31)
T3	12458	23	56713	1.41 (0.74-2.69)	1.37 (0.69-2.70)	1.60 (0.79-3.24)	0.86 (0.33-2.23)	1.20 (0.53-2.71)
			p-trend	0.29	0.38	0.20	0.73	0.53

BCAAs: Branched-chain amino acids; SUN: University of Navarra Follow-up; HR: Hazard ratio; CI: Confidence interval.

*Incident cases of T2DM.

1 adjusted for age (years) and sex.

2 additional adjusted for smoking status (non-smokers, current, former), pack-years of smoking (continuous), weight change in ≥+5 kg (yes/no), years of university education (continu-ous), hours of television viewing per day (continuous), family history of T2DM, physical activity (METs-h/week), Trichopoulou score tertiles, total energy intake (kcal/d), sugary beverage con-sumption (g/d), snacking between meals (yes/no), special diet (yes/no), prevalent hypertension (yes/no), prevalent cancer (yes/no), prevalent hypercholesterolemia (yes/no), prevalent hypertri-glyceridemia (yes/no), prevalent cardiovascular disease (yes/no).

3 additional adjusted for BMI (kg/m^2).

4 additional adjusted for protein intake (g/day).

5 repeated measurements after 10y.
